# A temperature-driven model for potato yellow vein virus transmission efficacy by *Trialeurodes vaporariorum* (Hemiptera: Aleyrodidae)

**DOI:** 10.1016/j.virusres.2020.198109

**Published:** 2020-11

**Authors:** H. Gamarra, P. Carhuapoma, L. Cumapa, G. González, J. Muñoz, M. Sporleder, J. Kreuze

**Affiliations:** a International Potato Center (CIP), Av. La Molina 1895, La Molina, Lima, Peru; b Agricultural Research Institute of Panama (IDIAP), Calle Carlos Lara 157, Panama; c National Agricultural University La Molina (UNALM), Av. La Molina 15024, La Molina, Lima, Peru

**Keywords:** Whitefly, Virus transmission, Modeling distribution with GIS, Temperature-based model, Virus distribution model, Potato diseases

## Abstract

Management of viral plant diseases can be improved by using models to predict disease spread. Potato yellow vein virus (PYVV) of the genus *Crinivirus (Closteroviridae)* is transmitted in a semi-persistent manner by the greenhouse whitefly *Trialeurodes vaporariorum* (Hemiptera: Aleyrodidae). Although several approaches exist for modeling insect population growth, modeling vector-born virus spread remains difficult because fundamental knowledge on the relationship between virus transmission and temperature is lacking for most vector transmitted viruses. To address this challenge, we initially developed a temperature-dependent phenology model for the whitefly vector using the Insect Life Cycle Modeling (ILCYM) software. In the present study, the effect of temperature on the efficiency of virus transmission by the whitefly was determined through controlled laboratory experiments at 8 constant temperatures in the range from 10 to 25 °C. The vector capacity to transmit the virus was highest at 15 °C (about 70 % probability of infection) but decreased radically as temperature deviated from this optimum temperature to <10 % at temperatures of 10 and 20 °C, respectively. The temperature-dependent probability of virus transmission by a single adult whitefly could be described by a nonlinear function, which was validated by transmission frequencies observed at fluctuating temperatures. This function combined with life table parameters calculated from previously established temperature-dependent phenology model for the vector provided a full temperature-responsive model for predicting PYVV spread potential and transmission probabilities. For spatial risk predictions, we devised two virus transmission risk indexes and tested their performance in correctly predicting virus presence/absence with field survey data. The best performing risk index was used to generate risk maps, which reflected well the current (real) occurrence of the virus but also predicted areas at high risk, where the virus has not previously been reported. One of them in western Panama was targeted for surveillance and resulted in identification of the virus in the country, where it was not previously known to occur. Simulated risk maps for the year 2050 revealed that climate change may significantly affect, the risk of distribution, generally reducing in tropical areas of the world, but increasing in the temperate regions.

## Highlights

• Transmission of potato yellow vein virus by a single whitefly vector was found to be highly temperature dependent.
• A non-linear mathematical function to describe the relationship between temperature and transmission likelihood was developed.
• A virus risk index was created by combining the virus transmission function and a whitefly phenology model.
• Detailed maps were generated indicating the risk of virus transmission using current and predicted future climate scenarios.
• Maps were used for surveillance of high-risk areas outside the known geographic range of the virus, leading to its discovery in a new region.

## 1 Introduction

The potato (*Solanum tuberosum* L.) is the fourth most important food crop worldwide, after wheat, rice and maize. The crop is affected by various pest and disease, among which viruses are one of the most important due to their gradual accumulation over generations of clonal propagation, eventually leading to yield decreases of 50 % or more. Global trade and a changing climate affect the distribution and impact of pests and diseases leading to emergence in new areas. Potato yellow vein disease, caused by Potato yellow vein virus (PYVV), a member in genus *Crinivirus* (family *Closteroviridae)* is an important disease of the crop, in Colombia, Ecuador and northern Peru, causing up to 50 % yield reductions (Guzmán-Barney et al., 2012; Salazar et al., 2000). PYVV is transmitted in a semi-persistent manner by the greenhouse whitefly *Trialeurodes vaporariorum* Westwood (Hemiptera: Aleyrodidae) (Lemma and Pulgarín Navarro, 1989; Salazar et al., 2000; Tamayo and Navarro Alzate, 1984), through tuber-seed, and underground stem-grafts (Alba, 1952; Salazar, 1996). Its origin has been traced to Northern Ecuador and the Central West Colombia region (Alba, 1952; Tamayo and Navarro Alzate, 1984) and since then the virus has spread throughout the Central Andes, particularly to the important potato-producing areas of Northern Peru (Salazar, 1996; SENASA, 2003) and all potato growing regions in the Andean highlands of Colombia (Franco-Lara et al., 2013; Guzmán-Barney et al., 2012; Guzmán et al., 2006; Rodríguez et al., 2015) and Venezuela.

PYVV is considered a quarantine pathogen by various agencies such as the European and Mediterranean Plant Protection Organization (EPPO) and the Animal and Plant Health Inspection Service (APHIS) (López et al., 2006) (USDA-APHIS, 2012). Potato, tomatoes and various weeds of *Polygonum* sp. (Polygonaceae) can act as reservoirs for PYVV (Salazar et al., 2000). As a result of climate change, the risk of geographical range expansion of PYVV is expected to alter as well. Due to the ectothermic nature of insects, changing temperature affects the distribution and abundance of the virus vector. Optimal growth and development of insects falls within a fairly broad range of temperatures. Within this optimal temperature range, increasing temperature leads to rising metabolic rates; in other words, increase development rates, escalating population growth and spread potentials of insect species and consequently related crop damages. A higher abundance of insect vectors implies increasing risks of virus transmission. The greenhouse whitefly, as the natural vector of PYVV, is predicted to expand its range to higher altitudes within the tropics and further in northern and southern latitudes due to increasing temperatures in the future (Gamarra et al., 2016). Thus, the whitefly might potentially transmit the virus to different hosts in new geographical niches, increasing the risk of virus spread (Salazar, 1998; Salazar et al., 2000).

Phenology/population models have become important analytical tools for understanding and predicting the growth potential and dynamics of insect populations under a variety of environmental conditions and management practices, and are proposed as helpful tools in pest risk assessment (PRA) (Kroschel et al., 2016). A temperature-driven phenology model for the greenhouse whitefly *T. vaporariorum* was presented in an accompanying publication of this issue (Gamara et al., accompanying manuscript). The model, which was implemented in Insect Life Cycle Modeling (ILCYM) software (Sporleder et al., 2013, 2017), predicts life table parameters of the species for fluctuating temperatures over time and risk indices for establishment and spread potential in space. ILCYM uses a deductive modeling approach by estimating the stage-specific development, survival and reproduction rates of the pest, with which it can calculate risk indices relating to pest establishment, activity and generations. ILCYM includes a Geographic Information System (GIS) environment that generates maps representing (predicting) the vector’s potential distribution and activity using the risk indices, either for the current or predicted future climate change scenarios. However, the model does not consider the effect of temperature on virus transmission efficiency. In fact, little data is available on the effect of temperature on virus transmission efficiencies by insect vector’s. Temperature, apart from affecting the vectors population growth and abundance, seems to influence the process (efficacy) of vector-born virus transmission as well, as has been demonstrated for mosquito transmission of Zika, Dengue and West Nile virus (Kilpatrick et al., 2008; Liu-Helmersson et al., 2014; Mordecai et al., 2017; Tesla et al., 2018). Also for plant viruses, few reports show temperature-dependence of transmission efficiencies by their insect vectors (Anhalt and Almeida, 2008; Bar-Joseph and Loebenstein, 1973; Lowles et al., 1996), although none of them attempted to determine a response curve. There are several key gaps that potentially affect the predictability of virus transmission rates for the PYVV-vector-potato pathosystem. For predicting accurately (the spread potential and risk of) yellow vein disease based on temperature we lack fundamental knowledge on the relationship between PYVV vector transmission and temperature.

The objectives of this study were *a)* to assess the effect of temperature on the PYVV transmission rates by its vector, the greenhouse whitefly, *b)* establish a temperature-dependent model for PYVV transmission probability by its vector and *c)* combine this model with the previously established *T. vaporariorum* phenology model for predicting the risk of virus spread based on temperature on global, regional, or national scales. The virus transmission model was implemented as an additional module in the ILCYM software. Thus, we demonstrate an approach that extends the knowledge gained from insect phenology models to assess risks of diseases transmitted by insect species in cultivated crop.

## 2 Materials and methods

### 2.1 Plant material, virus source and whitefly colonies

Virus–free potato plants (Clon W.A 077: CIP397077.16 and cultivar Canchan INIA: CIP380389.1) were obtained from the International Potato Center (CIP) germplasm bank in Lima, Peru.

PYVV was obtained from the CIP potato virus collection and was propagated and maintained in potato. Plants grown from infected tubers served as source plants for the transmission experiments.

For virus acquisition in the transmission experiments, two PYVV-infected source potato plants (grown from infected tubers) of the cultivar ‘Canchan INIA’ were planted in a temperature-controlled growth chamber per temperature tested. The temperature in the growth chamber was maintained at the same temperatures as used in the transmission experiments described below. Virus infection was confirmed approximately 25 days after planting by RT-PCR. In addition, properly sprouted healthy potatoes to be used as targets in inoculation experiments, were planted 10 days before virus transmission testing in growth chambers at the same temperature as used later in each transmission experiment.

A colony of *T. vaporariorum* originating from *Lantana camara*, but maintained on potato plants for 5 years at the institutes own rearing facility (greenhouse) at a temperature between 20−23 °C, 70–95 % RH and a photoperiod of 12:12 h light (L): dark (D), was used in the experiments.

Additional plant samples were collected from fields in Peru, Ecuador and western Panama and are described in section 2.6 below.

### 2.2 Virus transmission experiments

A diagram summarizing the virus transmission experiments is provided in Supplementary Fig. 1. PYVV transmission by *T. vaporariorum* was determined at 8 constant temperatures of 10°,12°, 14°, 15°, 16°, 18°, 20°, and 25 °C (±0.5 °C). For each temperature, 50 single whitefly transmissions (described below) were performed per replication and replicated 2 times per temperature initially. Based on results, 2 more replications were performed to assess the variation in results at key temperatures (12°, 14°, 15° and 20 °C). Whitefly transmissions were performed with an acquisition access period (AAP) to acquire the virus from the infected plants and inoculation access period (IAP) to transmit acquired virus to target plants (see diagrammatical representation in Supplementary Fig. 1). During the AAP, PYVV-infected potato plants (1 plant per 100 insects) were placed inside an acrylic box, covered with nylon mesh, in a growth chamber under each of the 8 predetermined temperatures. Adult whiteflies were caged with a clip-cage (17 × 13 × 13 cm) covered with nylon mesh to two leaves per plant (50 adults/leaf, one from the apical part and one from the middle part of the plant and were allowed to feed on the plants during this 48-h AAP. After this period, each of these adults were transferred individually directly to a single 10-day old healthy plant for a 72-h IAP covered by nylon mesh in growth chambers at the same temperature as during the AAP. Each batch of 50 insects that acquired the virus from one leaf was considered a replication. Since virus titres may vary in different parts of the plant and this may affect virus transmission, relative virus titres were determined from each leaf on which whiteflies had fed. To do this, the PYVV infected source leaves were harvested and stored at −80 °C for posterior quantitative analysis of PYVV RNA using RT-qPCR (section 2.3) directly after the AAP.

Each replication (replicated batch) consisted of 50 + 2 plants; 50 plants were exposed individually to a single whitefly that had previously an AAP as described above and 2 plants were controls in which the AAP was performed on uninfected plants. After the 72-h IAP, the insects were removed from the cages and the plants treated with the insecticide Applaud (Buprofezin) (Bayer) at 0.1 % to ensure that no insects remained. The inoculated plants and were subsequently maintained in a greenhouse at a photoperiod of 12:12 h L: D and temperature of 18° and 23 °C during the light and dark period, respectively for 35 days. After this 35-d incubation period, virus presence was assessed taking systemic leaves (non-inoculated leaves) submitted to RT-PCR analysis (section 2.3).

Separately, to validate the predictions of the temperature dependent transmission function (section 2.4 below) we used the methodology described above for constant temperatures, under fluctuating natural temperature conditions in a field exposed screen house in La Molina (Lima) during three distinct seasons at Lima, Peru, but with fewer (6) insects. In addition, insects were serially transferred every 24 h to new healthy potato plants for 10 days as long as the insects lived to determine i) transmission at as many as possible fluctuating temperatures for IAP (during different 24 h periods), and ii) to determine the period of infectivity for PYVV.

### 2.3 Reverse transcription polymerase chain reaction (RT-PCR), quantitative RT-PCR (RT-qPCR) and Nucleic Acid Spot Hybridization (NASH)

Total RNA was extracted from sampled leaves using Trizol® reagent (Invitrogen, Carlsbad, CA) according to the manufacturer's instructions. The cDNA for RT-qPCR was synthesized as follows: 7 μl of nuclease-free water (NFW), 2 μl of Random primers (250 ng/μl), 1 μl of dNTPs (10 mM) and 2 μl of total RNA (500 ng/μl) were mixed and denatured in a thermocycler (Applied Biosystems, Foster City, City, CA) at 65 °C for 10 min and then cooled to 10 °C for 5 min.

Subsequently, RT Mix (4 μl 5X First Strand Buffer, 2 μl DTT 100 mM, 0.5 μl RNAse OUT and 0.5 μl M-MLV reversed transcriptase) was added and then incubated at 37 °C for 50 min, followed by 70 °C for 15 min and finally cooled to 10 °C. The final volume obtained was a 20 μl cDNA, which was diluted with 80 μl of NFW to obtain a dilution of 1:5 for subsequent PCR.

Relative PYVV concentrations in source leaves from each temperature were assessed to determine if temperature and leaf position had any effect on virus titres, that could influence transmission results. The PYVV primers (qPYVV_F_CP, qPYVV_R_CP) targeted the capsid region of the virus and the Cytochrome oxidase I (COX) gene was used as a reference gene (Supplementary Table 1).

The reaction mixture was prepared in a total volume of 10 μl containing: 4 μl of c-DNA (50 ng), 0.4μM of each primer, and 5μl PCR Master Mix Power SYBR Green (Applied Biosystems StepOne Real-Time PCR System).The following PCR program was used: PCR: 95 °C for 10 min, and then 35 cycles at 95 °C for 10 s, 50 °C for 45 s, 60 °C for 1 min, and finalized with a melting curve analysis. Fluorescence was measured after each amplification cycle. *Ct* values were determined using Applied Biosystem StepOne version 2.3 software. Samples were considered positive when they reached the *Ct* value before the 35^th^ cycle. Each sample was performed in three technical replicates, including infected and healthy controls. Blanks were included in each separate PCR run. Data was analyzed using the 2^(−ΔΔ^*^Ct^*^)^ method (Livak and Schmittgen, 2001) to determine relative RNA levels and expressed as compared to the lowest value obtained, which was set at 1.

To verify successful virus-transmission by each individual whitefly to healthy potato plants as well as field samples from Panama (see section 2.6), regular RT-PCR was used, with primers PYVV CP1_F and PYVV CP2_R that amplified a 760 base pair fragment of the CP gene (Supplementary Table 1). The reaction conditions were as follows: 4 μL of PCR 5X Buffer (Promega), 1 μl of MgCl2 (25 mM), 0.5 μl of dNTPs (10 mM), 0.5 μL of Primers (CP1_F / CP2_R) both at 10 μM, 0.5 μl of the Taq polymerase (Promega), 8 μl of nuclease-free water (NFW) and 5 μl of diluted cDNA. The mixture was placed in a thermal cycler with the following conditions: one cycle at 94 °C for 5 min, followed by 35 cycles at 94 °C for 1 min, 56 °C for 1 min, and 72 °C for 1 min followed by 10 min at 72 °C. Amplified bands were visualized in a 1% agarose gel stained with Gelred (Invitrogen) under UV light.

Samples from field surveys in Peru and Ecuador were tested for PYVV by nucleic acid spot hybridization (NASH). Two ½ leaflets were macerated in 2 ml, of 5x SSC (saline-sodium citrate) buffer in 100 μm polyethylene plastic bags (4 × 6 cm) and 30 μl of crude leaf sap was blotted on a nylon membrane (Hybond-N; Amersham Biosciences AB) dried and cross-linked by UV-irradiation (50 mJ) in a cross-linking oven (Stratagene). Each membrane also included extract from a positive PYVV infected and healthy control plant of cv. Canchan INIA. Digoxigenin (DIG)-labelled probes encompassing the coat protein gene region (CP) of PYVV were synthesized by PCR as described above using primers PYVV CP1 and PYVV CP2 (Salazar et al., 2000), and DIG-labelled dNTPs (Roche, West Sussex, UK). The spotted and cross-linked membranes were prehybridized for 30 min at 55 °C in DIG Easy Hybridization buffer and then hybridized in the same solution at 55 °C for 16 h after adding the DIG-labelled probe. After hybridization, membranes were washed twice in 2X SSC and 1% SDS at room temperature for 15 min, incubated for 30 min with anti-DIG antibodies conjugated with alkaline phosphatase, and washed twice with maleate buffer with 0.3 % Tween-20. The reaction was developed using CSPD chemiluminescent substrate (Roche) and Omat-S film (Kodak).

### 2.4 Developing the mathematical model for virus transmission efficiency

The transmission as evaluated for each adult in a $n_{j}$ sample at the *j*^th^ temperature $T_{j}$, was considered as a dichotomous variable *y*_*ij*_, with values of 1 for success and 0 for failure, for the I^th^ insect at the j^th^ temperature to obtain the absolute frequency, or total number of insects that transmitted the virus for every temperature $\sum_{i}^{n}y_{ij}$, and finally to obtain the percent transmission $\;p_{j}=\frac{\sum_{i}^{n}y_{ij}}{\;n_{j}}$. In that way, a value for the transmission rate was obtained for every constant temperature assessed, where the temperature is the independent variable, and the percentage of transmission is the dependent variable; this relationship is represented by a nonlinear function $\;f\left(\text{T}\right)=p$.

We tested about 20 models available in ILCYM that were developed for describing insect development rates, and the best models were selected according to AICc. Parameters for each of these models are automatically estimated by ILCYM according to the Levenberg-Marquardt algorithm (Moré, 1978) using the function “nls.lm” (contained in the R package “minpack.lm”), which minimizes the sum of squares of the vectors returned for each function. Several of these models showed a significant overall fit, and the we initially used the Janish model for surveillance guidance (see results). However, we felt that none of these enzyme-kinetics-ruled functions described the data accurately. Therefore, we subsequently developed and tested additional models that assume exponential or double exponential reduction in transmission efficiency as temperature deviates below or above of an optimal temperature, respectively (see results section).

All candidate models were implemented as optional functions in a new tool within the ILCYM package for analyzing temperature-dependent vector virus transmission probability (efficiency) in insect vectors. All statistical calculations and coding for model implementation were made in R-3.4.1 (R Core Team, 2017).

### 2.5 Developing a risk index for the virus

We tested two alternative methods to establishing a PYVV risk index based on the temperature-dependent virus transmission efficiency combined with the phenology model established previously for the vector (Gamara et al., 2020, accompanying manuscript). The temperature based phenology model predicts *T. vaporariorum* temperature-dependent life table parameters–namely net reproduction rate (*R_0_*), mean generation time (*T*), intrinsic rate of natural increase (*r_m_*), finite rate of increase (*λ*) and doubling time (*D_t_*), based on monthly minimum and maximum temperature data. They provide useful information about the expected vector abundance in a given environment. The two indices were developed combining the information on virus transmission efficiency and the vectors life table parameters.

The first index, “virus transmission index (VTI) 1″, is based on the virus transmission probability and the whitefly’s finite rate of population increase. The finite rate of increase, *λ*, defines the average per-capita multiplication factor per time-step (in this study per month); it means, populations are expected to grow–assumed that the population has reached a stable age-stage distribution–by the factor *λ* each month. Thus, during a given time interval of *x* months a population of the size *N_i_* grows by the factor *λ*^x^.

$$N_{i+x}=N_{i}{\times \lambda }^{x}$$

Therefore, the growth rate *λ* seems an adequate factor for projecting relative spatial or seasonal differences in field densities (abundance) of pest populations, assuming that the initial population size is equal among space or at the beginning of a season. The temperature-dependent vector transmission efficiency factor, *p*, as described in the previous section, defines the probability that a single virus-carrying vector transmits the virus to the plant after attacking the plant. Since knowledge about the biological processes for successful virus transmission and disease development in virus-inoculated potatoes is lacking, we favored a non-additive model, in which each vector that has acquired the virus has the same chance of causing an infection in the plant after feeding for a given time interval on the host plant. The attack (contact) rate is directly proportional to the density of the vector. If *p* is the vector’s probability of inducing a virus infection after a given feeding period, the probability that the plant will be not infected at vector densities of *x* vectors per plant is:

(2) $$Q_{x}={\left(1-p\right)}^{x}$$

and the probability of infection, *P*, is

(3) $$P_{x}={1-\left(1-p\right)}^{x}$$

We considered the vectors life table parameter, *λ*, as a useful indicator for a reasonably representation of relative differences in vector densities among different locations or between specific seasons in a given location in regard to environmental temperature. Accordingly, for our risk index we exchanged the parameter for vector densities in eq. [1] with the temperature-dependent life table parameter *λ*. The risk index for a given month in each location is calculated as follows:

(4) $${VTI1}_{i}={1-\left(1-p_{i}\right)}^{{l_{i}}^{\omega }}$$

where, *VTI_i_* is the virus transmission risk index for the *i*^th^ month, *p_i_* is the temperature-dependent virus transmission efficiency rate during the *i*^th^ month (as described in the previous section), *λ_i_* is the temperature-dependent finite rate of increase of the vector population, and ω is a shape factor (we tested ω = 1–35 to evaluate which achieved the best prediction based on survey results). The index values are bound between 0 and 1. The annual virus transmission risk index for given location is calculated as:

(5) $${VTI1}_{Annual}=\frac{\sum_{i=1}^{12}{VTI}_{i}}{12}$$

The second index VTI2 is a simple index, calculated by averaging the whitefly’s monthly net reproduction rate, *R*_0_, multiplied with the temperature-specific virus transmission efficiency, *p*, over a year. The equation was:

(6) $$\;{VTI2}_{Annual}=\frac{\sum_{i=1}^{12}R_{0_{i}}\times p_{i}}{12}$$

where *R*_0_*_i_* and *p*_i_ are the net reproduction rates and the virus transmission probability, respectively, predicted for the *i*^th^ month.

To evaluate the predictive capacity of VTI1 and VTI2 we used a dataset containing 817 geo-referenced virus presence/absence data derived from a field surveys performed in Colombia, Ecuador and Peru during 2007–2010 (see section 2.6). Only locations where whitefly presence had been confirmed were used (Supplementary Fig. 2). Contingency tables were established between real and expected (based on year 2000 climate data) PYVV presence or absence using variable index threshold values. The correct prediction of real virus presence and absence revealed from the data set was plotted against the simulated indices VTI1 and VTI2 (the prior was tested with different settings for the parameter ω). To illustrate the predictive ability of the index as a binary classifier for virus presence the performance of each index was tested by plotting the true positive rate (TPR = sensitivity) and the true negative rate (specificity) against various threshold settings of the index as well as receiver operating characteristic (ROC) curves (Metz, 1978) by plotting the TPR against the false positive rate (1-specificity). The index that revealed the higher area under the ROC curve was implemented in the geographic simulation module in ILCYM software using R coding for generating risk maps base on this index.

### 2.6 Field surveys

Geo-referenced field surveys were performed to assess the presence of *T. vaporariorum* and PYVV in potato crops in Peru (1788 fields) and Ecuador (3 fields) between 2007–2010 (Supplementary material 1). Depending on the size of the fields, a number of 10–20 plants were evaluated. The presence or absence of PYVV symptoms were recorded for each plant on fully expanded leaves. Collected leaves were stored in a Styrofoam box filled with ice and later (at the same day) plant sap blotted on nylon membranes and air dried for virus-detection tests using NASH as described in section 2.3. Whitefly presence was evaluated by examining plants visually, and if whiteflies were found, pupae were collected from the plants for posterior species identification by morphology under stereoscope in the lab. Additional data from field surveys performed in Colombia (62 fields) during 2008 (Franco-Lara et al., 2013) were included.

Subsequently, to evaluate the usefulness of the model to guide surveillance, we identified an area with significant risk, based on the selected VTI, for establishment and spread of PYVV in western Panama and surveyed the area for presence of whiteflies and symptoms corresponding to virus infection during 2016. Leaves from 26 potato plants were collected in 3 potato fields to verify the presence of PYVV, and identify the whitefly species from nymphs. The fields were located at Cerro Punta: (8° 51′ 20″ N of Latitude and 82° 32′ 45″ W Longitude; 10 samples); Las Nuves (8° 52′ 1″ N and 82° 7′ 49″ W; 15 samples) and La Gavidia (8° 51′ 56″ N and 82° 35′ 27″ W; 11 samples). The samples were tested with RT-PCR using primers specific for PYVV as described above.

### 2.7 Generation of risk maps and climate data used

Maps were generated using ILCYM, which works with monthly maximum and minimum temperature data sets for one year. Twelve months data sets with their respective geographical coordinates were obtained at a resolution of 10 min for world prediction and 2.5 min for the region between South America and Central America from the WorldClim database (www.worldclim.org)and converted to *.flt* (float type) data format. For future predictions the temperature database with the RCP 6.0 scenario and the CCSM model were used. The extracted temperature data were organized in 12 × 2 matrices using the longitude as columns and latitude as rows representing 12 matrices each for the minimum and maximum temperatures. Thereafter, a point object was created for each geographical coordinate (longitude and latitude) in form of a two-column table (the first column included the minimum temperature and the second the maximum temperature for each point) that was used directly for spatial phenological simulation (for further details on these methods see Kroschel et al., 2013). These temperature data served as input data for calculating the Virus Transmission index (VTI) as, described in section 2.5 above, for each point object (cell) and according the resolution (dimension of the cell).

## 3 Results

### 3.1 Virus transmission model

**Fig. 1:**
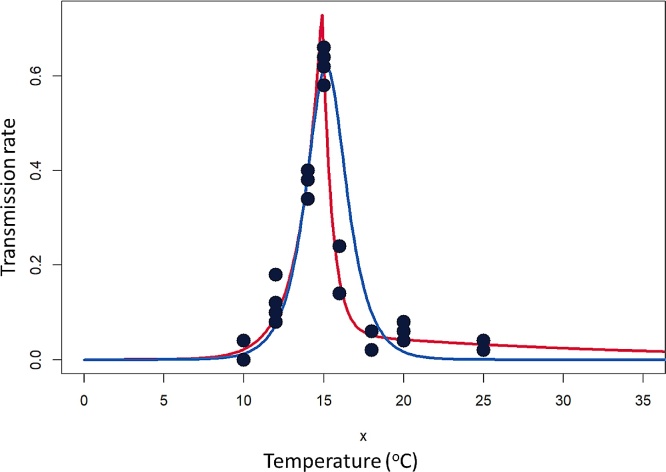
Mathematical function fitted to the temperature-dependent virus transmission rates, blue: modified Janish function available in ILCYM 4, red: new Sporleder 2 function (see Table 1 for function and parameters).

The efficiency of PYVV transmission by *T. vaporariorum* in potato was strongly dependent on temperature, with an optimal efficiency of 0.58 – 0.66 at 15 °C that rapidly dropped to less than 10 % below 12 °C or above 18 °C (Fig. 1). Detailed statistical results for fit among the available functions in ILCYM and the newly developed Sporleder 2 (see below) function are provided in Supplementary material 2. Among the originally available functions in ILCYM the modified Janisch model described best the relationship between virus transmission efficacy and temperature based on AICc

(7) modified Janisch model: (Janisch, 1932) $f\left(T\right)=p=\frac{{2TR}_{min}}{\left(e^{k\left(T-T_{opt}\right)}+e^{-k\left(T-T_{opt}\right)}\right)}$

where *TR*_min_ and *T_opt_* is the virus transmission efficiency rate (*TR*_min_) at the optimum temperature (*T_opt_*), and *k* is an empirical constant determining the shape of the function. The function explained >81 % of the variation in the data (Fig. 1, Table 1). This model was initially used for developing risk indices (Section 2.3) and risk maps (Section 2.4) to identify target areas for surveillance.

**Table 1:** Estimated parameters fitted to describe temperature-dependent virus transmission efficacy of PYVV by *T. vaporariorum* with the Janish (A) and Sporleder (B) models.

**A** Parameter estimates of the Janisch (1932) model^A^			*F*-value	*df*_1, 2_	*P*	*AICc*
*TR_min_*	*T_opt_*	*k*				
1.61 (±0.15)***	15.2 (±0.11)***	0.88 (±0.23)***	45.87	2, 21	<0.001	−37.380


**B** Parameter estimates of the Sporleder 2 model^B^			*F*-value	*df*_1, 2_	*P*	*AICc*
*TR_max_ T_opt_*	γ *k_low_*	*K_high1_ k_high2_*				

0.74 (±0.03)*** 14.88 (±0.05)***	0.07 (±0.02)*** 0.71 (±0.04)***	1.55 (±0.08)*** 0.05 (±0.26)*	159.64	5, 21	<0.001	−82.588

Numbers in parenthesis are standard errors. Parameter values significantly different from zero are indicated by asterisks (P < 0.05 = *, P < 0.01 = **, P < 0.001 = ***).

^A^The equation of the Janisch model (Janisch, 1932) is: $f\left(T\right)=p=\frac{{2*TR}_{min}}{\left(e^{k\left(T-T_{opt}\right)}+e^{-k\left(T-T_{opt}\right)}\right)}$ where *TR*_*min*_ and *T*_*opt*_ is the virus transmission efficiency rate (*TR_min_*) at the optimum temperature (*T*_*opt*_), and *k* is an empirical constant determining the shape of the function.

^B^The equation of the Sporleder 2 model is:*f(T)*=*p* = min(*TR*_*max*_(*e*^*-k*^_^low^_^[*T − Topt]*^), *TR*_*max*_([*1-γ*]*e*^[^*^-k^*_*^high1^*_*^-k^*_*^high2^*_^]^(*T − T*_*opt*_)+γ*e *^*− k*^_^high2^_^[*T − T*^*opt*^]^)) where *TR_max_* is transmission rate at the optimum temperature, *T_opt_*, and *k_low_* and *k_high1_* and *k_high2_* are the decay constants at low and high temperature, respectively. The temperature change (either temperature increase above *T_opt_* or temperature reduction below *T_opt_*) causing 50 % reduction in the transmission rate (*T*_½_) can be written in terms of the decay constants as: *T*_½_ = *k_low_* * ln (2) and *T*_½_ = *k_low_* * ln (2), respectively.

However, we felt that the function did not describe well the asymmetric distribution of the data and so we subsequently developed additional functions for which the equations were as follows:

(8) $$\text{S}\text{p}\text{o}\text{r}\text{l}\text{e}\text{d}\text{e}\text{r}-1:TR\left(T\right)=\left\{\begin{array}{c} {TR}_{max}\;\times \text{e}\text{x}\text{p}\left({-k}_{low}\left[T_{opt}-T\right]\right)\\ {TR}_{max}\times \;\text{e}\text{x}\text{p}\left({-k}_{high}\left[{T-T}_{opt}\right]\right) \end{array}\right)\;\begin{array}{c} if:T<T_{opt}\\ if:T>T_{opt} \end{array}$$

where *TR_max_* is transmission rate at the optimum temperature, *T_opt_*, and *k_low_* and *k_high_* are the decay constants at low and high temperature, respectively. The temperature change (either temperature increase above *T_opt_* or temperature reduction below *T_opt_*) causing 50 % reduction in the transmission rate (*T*_½_) can be written in terms of the decay constants as: *T*_½_ = *k_low_* * ln (2) and *T*_½_ = *k_low_* * ln (2), respectively.

(9) $$\text{S}\text{p}\text{o}\text{r}\text{l}\text{e}\text{d}\text{e}\text{r}\;2:TR\left(T\right)=\left\{\begin{array}{c} f_{1}\left(T\right)={TR}_{max}\;\times \text{e}\text{x}\text{p}\left({-k}_{low}\left[T_{opt}-T\right]\right)\\ {f_{2}\left(T\right)=TR}_{max}\left(\left[1-\text{γ}\right]\text{e}\text{x}\text{p}\left(\left[{-k}_{high1}-k_{high2}\right]\left[\text{T}-T_{opt}\right]\right)+\text{γ}\;\text{e}\text{x}\text{p}\left({-k}_{high2}\left[{T-T}_{opt}\right]\right)\right) \end{array}\right)\;\begin{array}{c} if:T<T_{opt}\\ if:T>T_{opt} \end{array}$$

which assumes a double exponential decay function above the optimum temperature. In the double exponential decay model it is assumed that two processes affect virus transmission; 1) due to increasing temperature virus transmission decays fast by the constant *k_high1_* into a state at which their activity (probability to be transmitted and cause infection in the plant) is reduced to γ, and 2) virus transmission of all states are reduced by a slower decay constant determined by *k_high2_*. Based on the conditions of the model and its application in terms of a function, the Sporleder 2 model can be defined as follows:

$$f\left(T\right)=p=min\left(f_{1}\left(T\right),f_{2}\left(T\right)\right)$$

The Sporleder 2 function was 277 million times better than the modified Janish according to AICc evidence ratios (AICc modified Janish: 33.35, AICc Sporleder 2: -23.98)

To reveal if a relationship exists between virus transmission and virus titres in the source plants

at different temperatures, we performed qRT-PCR analysis from all source leaves used in the transmission experiments.

The transmission efficiency had no correlation with virus concentration (correlation coefficient = -0.31) in the plant as determined by qRT-PCR, as virus titres did not vary much between 10° to 18 °C, but then increased significantly at temperatures above 20 °C (Supplementary Fig. 3), where transmission was inefficient (Fig. 1). The relationship between virus titre and temperature was better described by an exponential function (AIC 473.06; residual standard error 467.7) than a linear one (AIC 480.67; residual standard error 528.7).

The screenhouse experiment in La Molina for testing virus transmissions at naturally fluctuating temperatures during 3 seasons: February (summer), May (autumn), July (winter) were in agreement with the established virus transmission model. Only during July, that was the period in which temperature frequently fell below 17 °C at which efficient virus transmission was expected, plant exposure to vectors resulted in successful virus transmission. During the other season, temperature remained >17 °C, which was above the expected temperature limit for efficient virus transmission, and no transmission was observed (Supplementary Table 2).

### 3.2 Performance of virus transmission risk indices

**Fig. 2:**
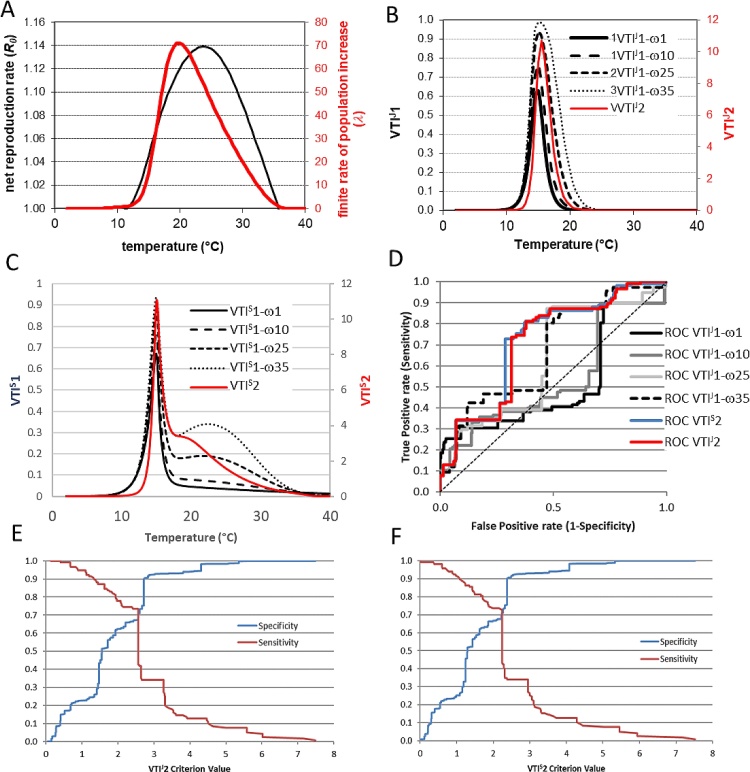
Comparing different virus risk indices based on the expected temperature-dependent virus transmission efficiency rate combined with selected life table parameters of the vector (i.e. the finite rate of population increase, λ, and the net reproduction, *R_0_*); A: performance curve of the life table parameters λ and *R_0_*, established earlier (see Gamarra et al., 2020); B: predicted risk indices, VTI^J^1 with variable scale parameter ω and VTI^J^2 in relation to temperature; C: risk indices, VTI^S^1 with variable scale parameter ω and VTI^S^2 in relation to temperature; D: resulting ROC curves of the indicated indices generated for the virus presence/absence survey data, E-F: resulting true positive rate (sensitivity) against the true negative rate (specificity) curves for VTI^J^2 (E), VTI^S^2 (F).

The risk indices were evaluated using both the original modified Janish function and the Sporleder 2 function developed in this study and are henceforward referred to as VTI^J^and VTI^S^ respectively, whereas VTI applies to the risk indices in general. The differences between the indices VTI1 and VTI2 is based on the expected temperature-dependent virus transmission efficiency and the vector’s two life table parameters, either the finite rate of population increase or the net reproduction rate are illustrated in Fig. 2a. VTI1 with a scale parameter setting ω = 1 almost corresponded with the expected temperature-dependent virus transmission efficiency rate, while increasing scale parameter settings increase the risk estimates toward higher temperatures (see Fig. 2b & c). In comparison, VTI2 indicates a shallower risk curve corresponding to the virus transmission efficiency curve shifted upward on the temperature scale. The main differences between VTI^J^ and VTI^S^ indices was the narrower range of optimal temperatures in VTI^S^ and a lower but broader secondary peak at higher temperatures in VTI^S^ (Fig. 2b & c). The secondary peak is a result of optimum of finite population increase and net reproduction rates (both being at higher temperatures then the estimated transmission efficiency) influencing the curve shape of the VTI^S^ at temperatures above 20 °C because the estimates for the Sporleder 2 are >0 in that range in contrast to the modified Janish function.

VTI2 performed better than VTI1 at any scale parameter setting when tested against the real virus presence/absence data sampled during the survey (Supplementary material 1). VTI^J^2 revealed the highest area under the ROC curve (AUC) of 0.71, while the highest AUC of 0.67 was obtained using VTI^J^1 with ω = 35 (Fig. 2d). Therefore, VTI^J^2 was initially chosen as the more accurate index for risk mapping and used to guide surveillance in Panama (section 3.3). We subsequently developed the Sporleder 2 function and applied it to VTI1 and VTI2. VTI^S^2 had a slightly improved AUC of 0.72 as compared to VTI^J^2, whereas the highest AUC for VTI^S^1 was 0.64 also with ω = 35. The sensitivity (true positive rate) vs specificity (true negative rate) plot for the field survey data for VTI^J^2 and VTI^S^2 are illustrated in Fig. 2e and f and were almost identical in shape. Applying the point of crossing of the specificity and sensitivity curves to obtain the optimal tradeoff of true positive and negative predictions resulted in critical thresholds of 2.56 and 2.23 for VTI^J^2 and VTI^S^2 respectively and resulted in the best overall prediction accuracy of 70.4 % for VTI^S^2 (Table 2) whereas it was 68.4 % for VTI^J^2. At this threshold value 70.9 % of the true virus presence (sensitivity) and 72.1 % of the true absence (specificity) were correctly predicted, revealing a precision (positive predictive value) of 29.0 %, while the negative predictive value was 93.9 %. The low positive predictive value is due to the unbalanced occurrence of the virus in the sampled locations (PYVV was detected in 10.2 % of the surveyed locations only).

**Table 2:** Accuracy of prediction using VTI^S^2 > 2.23: ((86 + 493)/822) *100 = 70.4 %.

	Presence	Absence
**Estimated presence**	86	211
**Estimated Absence**	32	493
	**Total**	822

The F1 score, as a measure of the threshold accuracy considering the tradeoff between precision and the true positive rate, (sensitivity) revealed a value of 0.516 for a VTI^S^2 threshold of 2.23. In contrast, VTI1 with variable parameter settings of ω revealed low F1 scores (not shown).

### 3.3 Risk maps for PYVV establishment and spread

**Fig. 3:**
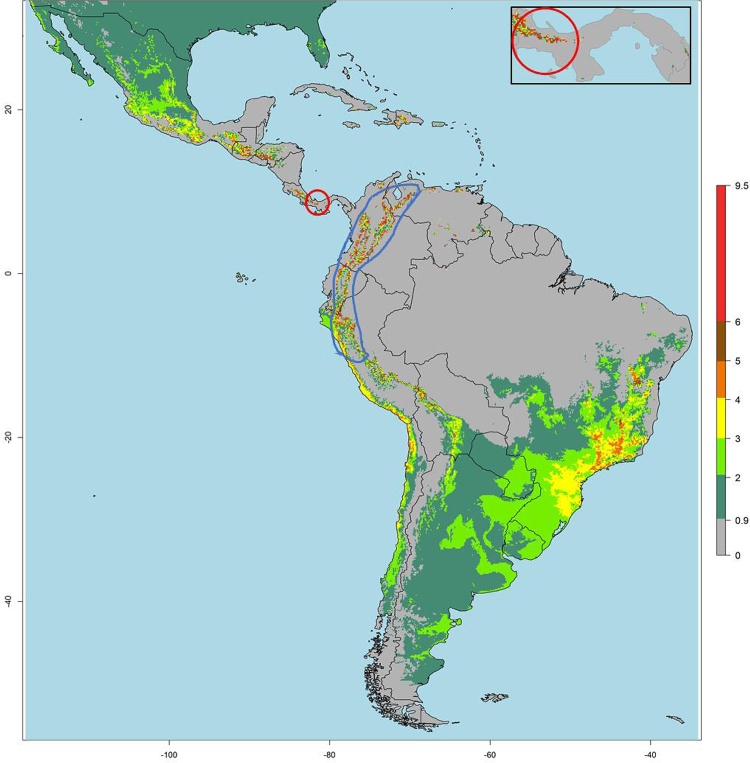
Predicted VTI^J^2 risk for PYVV by *T. vaporariorum* in Latin America for the year 2000. The blue line indicates the area in which the virus was known to be endemic before this study and the red circle in Western Panama indicates a region predicted at high risk for virus transmission and was visited for surveillance confirming the presence of the virus. The region is enlarged in the inset.

To test the effectiveness of using the VTI2 maps for guiding surveillance, we used VTI^J^2 and year 2000 climate data (the most recent data available in 2015) and identified a region in western Panama that was predicted to have high VTI^J^2 (red circle in Fig. 3), whereas PYVV had not yet been reported in this country. We targeted this region for sample collection in 2016. Potato plants and weeds with symptoms typical of PYVV could be seen in all three potato fields visited in Panama (Supplementary Fig. 4). The result by RT-PCR revealed 50 %, 20 % and 18 % of the sampled plants were infected with PYVV in the three surveyed fields (Supplementary table 3). Also the vector *T.vaporariorum* was present in two of the three fields sampled (Station IDIAP and La Gavidia).

Subsequent to this effort the new Sporleder 2 function, which better fit the transmission data, was developed based on which new VTI^S^2 maps were generated. VTI^J^2 and VTI^S^2 maps were very similar except that areas with higher annual average temperature (between 18−30 °C) increased in their risk predicted by VTI^S^2, but were still low, and areas of the highest risk were reduced (Supplementary Fig. 5).

**Fig. 4:**
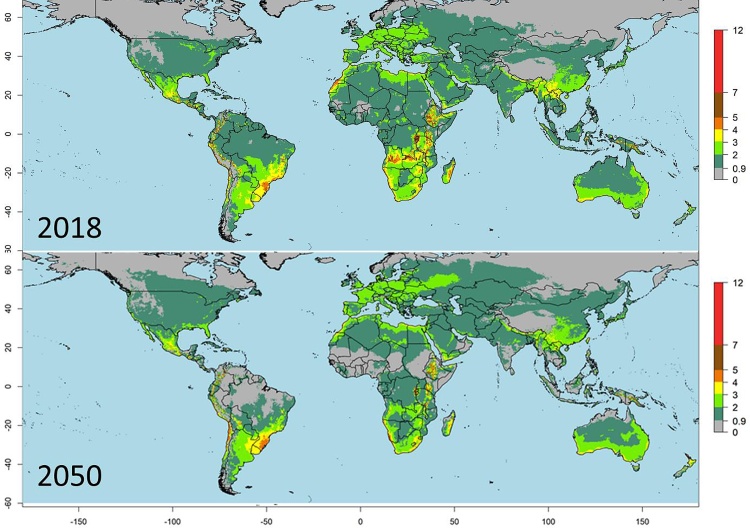
Virus Transmission Index 2 based on the Sporleder 2 transmission function (VTI^S^2) maps for PYVV spread by *T. vaporariorum* for the years 2018 and 2050.

Global risk maps were created using the VTI^S^2 indices using current and future predicted annual climate data, revealing current and future areas at significant risk of PYVV epidemics (Fig. 4). Based on the analysis in described in section 3.2, regions where the VTI^S^2 > 2.23 indicate temperature conditions where the combination of *T. vaporariorum* population growth potential and their virus transmission efficiency on an annual basis represent a significant risk for establishment and spread of PYVV. The maps reveal that under current climate conditions in South America, where PYVV is already present in Panama (as determined in this study), Colombia, Venezuela, Ecuador and Peru, that there are areas at risk of further spread and establishment in Costa Rica, Bolivia, Chile and Argentina as well (Fig. 4; Supplementary Fig 5). Under the year 2050 temperature scenario, the risk increases in the mountainous regions where PYVV is already present and is predicted to have a potential to spread further to the southern regions of Peru, Bolivia, Chile and Argentina. The VTI2 will increase at higher latitudes, and altitudes in the tropics in the future, whereas it will decrease in the lower altitudes in tropical regions (Fig. 4). These areas are important for the production of food crops such as potato and tomato, which are both host of PYVV.

## 4 Discussion

This study provides experimental data showing that the transmission of PYVV by individual adults of *T. vaporariorum* is highly temperature dependent with a narrow range of efficient transmission between 12 and 18 °C. Under optimal temperature conditions (∼15 °C) it can be a highly efficient vector with a transmission rate of 0.58−0.66 per individual insect. Strikingly, the transmission efficiency had no correlation with virus concentration in the plant as determined by qRT-PCR, as virus titres did not vary much between 10° to 18 °C, but then increased significantly at temperatures above 20 °C (Supplementary Fig. 3), where transmission was inefficient (Fig. 1). This suggest that other factors, possibly relating to vector behavior (in probing and or feeding habit) or interactions between specific virus, vector and host proteins required for transmission may be involved. Because both acquisitions and transmission were performed at the same temperature, it is also unclear if the variable transmission is a result of an effect on virus acquisition or transmission by the vector. Studies in other virus vector systems have shown that temperature may affect both of these processes differently (Anhalt and Almeida, 2008). Follow up studies to establish the exact mechanism of temperature influence on vector transmission efficiency should be an interesting field of future investigation.

Although several functions among those generally used for describing temperature-dependent development in insects available in ILCYM showed a significant overall fit to describe the relationship between temperature and virus transmission of PYVV by *T. vaporiarum* (p < 0.05, e.g. Janisch model; Supplementary material 2; Table 1, Fig. 1), our own developed functions performed better (confirmed by AICc’ evidence ratios). It seems that the virus transmission efficacy cannot be explained well by the enzyme-kinetic-describing type of models even after modifications. The best model according to AICc’ evidence ratios was the new Sporleder-2 model which proposes a biologically meaningful theoretical explanation of the process involved in PYVV-transmission: the probability of successful transmission decreases exponentially as temperature deviates from the optimum temperature with different decay constants above and below the optimum temperature of about 15 °C. While at temperature below the optimum the transmission efficiency decreases at a constant rate (each decrease of 1.2 °C in temperature decreases the probability of infection by 50 %; henceforth refereed to half-life temperature, T½), at temperature above the optimum, the decay in transmission efficacy is explained by two-components; with increasing temperature 1) initial transmission efficacy decreases at a relative high rate (T½ =0.48 °C) to a level at which the probability of infection is about 5.7 % (this can be explained by a conversion of the virus into a second state at which it retains only 5.7 % the initial transmission activity), and 2) transmission efficacy decreases by a second slower rate at higher temperatures (at about 17 °C). The latter can be explained by inactivation of virus transmission of both states (active virus and virus of reduced transmission activity) due to high temperature at a relative slow rate (T½ = 12.8 °C). However, the curve would look the same if the efficiency of causing an infection of active virus particles increases again at high temperature. Therefore the exponential increase in virus titres we observed from our qRT-PCR data (Supplementary Fig. 3) could also explain the flatter course of the transmission curve above 17 °C. To validate the models, transmission experiments were performed under naturally fluctuating conditions during three different seasons in la Molina. In only one of these seasons was the temperature predicted to be within the range that efficient virus transmission could take place, and this was confirmed in the experiments (Supplementary Table 2). In the winter season where transmission did take place, the actual efficiency was higher than that predicted by the model based on results under controlled conditions, even if this result was based on only very few observations and therefore not statistically powered.

When comparing the function describing the transmission (Fig. 1), to functions describing aspects of the insect phenology (Fig. 2a), it was clear that the optimal temperature for the vector development and virus transmission were not overlapping. The implication of this is that models simulating insect development as a proxy for risk of virus transmission will provide highly inaccurate results, as the optimal temperature for insect population growth coincides with a transmission efficiency of only around only 3% or less. To address this problem and attempt to generate more meaningful maps for virus risk, two risk indexes, VTI1 and 2 were devised, which considered finate rate of increase and net reproduction rate of the whitefly respectively. VTI1 is more complex but includes a shape factor (ω), which can be modified to adjust the curve to the data. Nevertheless, various rounds of testing with different ω factors, including with the two alternative transmission functions *f*(T) for VTI1 and 2, always resulted in the simpler VTI2 function generating better predictions based on testing with the field survey data. VTI2 generates risk indices with values ranging from 0 to ∼11. The maximum accuracy of correctly predicting virus presence and absence using our field survey data was 70.4 % % using a VTI^S^2 of 2.23 as a cutoff (Table 2) even if overall results for VTI^S^2 and VTI^J^2 were generally very similar (Fig. 2 d–f, supplementary Fig 5). We consider this a good correlation, bearing in mind that the virus can also be transmitted by infected seed tubers, which is likely to be a major contributor to virus spread and could cause virus to be present in areas not due to vector transmission (Cuadros et al., 2017; Guzmán-Barney et al., 2013).

Applying either VTI2 to generate global risk maps revealed a remarkable coincidence of predicted high risk areas with the regions where the virus was known to be a problem within its current endemic range (Venezuela, Colombia, Ecuador and Peru; Fig. 3 & Supplementary Fig 5). Beyond the regions where PYVV is currently endemic, the maps showed high risk of virus spread (and thus establishment if the virus were introduced) in regions of Africa, with the Western lake Victoria crescent and Eastern rift mountains of East Africa showing a particularly high risk (Fig. 4). Countries within these regions should be particularly vigilant to this virus regarding the import of potatoes from countries where the virus is endemic. On the American continent areas predicted to have a high risk of virus spread by the vector included the Eastern and Western slopes of the Andes in Southern Peru, Northern Chile and Bolivia to the South, and the highland regions of central America, including western Panama to the North (Fig. 3 & Supplementary Fig 5). Because Panama borders Colombia where PYVV is endemic, we considered it might be at risk of introduction of the virus and a small survey was performed in potato growing regions predicted to be at high risk in 2016 (Fig. 3). This resulted in the confirmation of the presence of virus symptoms (Supplementary Fig. 4), the virus (Supplementary table 3) and its vector in this region, providing an example of how the generated maps can effectively support targeted surveillance strategies in areas identified to be at risk. Based on the predictions that western Panama forms a continuous area of high VTI2 with adjacent potato growing regions in Costa Rica it is likely that we would find presence of the virus in this country as well and should be a target for future surveillance. The highlands of Honduras, Guatemala and southern Mexico are also predicted to have a high risk of spread if introduced (Fig. 3, Supplementary Fig. 5) and these countries should be vigilant to the introduction of the virus through informal trade of potatoes, which is the most likely means it entered into Panama from Colombia.

A common way to generate predictions of pathogen/disease range is using occurrence records coupled with some correlative species distribution model or habitat suitability modeling. This ‘inductive’ modeling approach has advantages where detailed information about species is not available, however due to lack of consideration of species’ biological characteristics in the modeling framework (Venette et al., 2010) resulting risk maps may inform about potential establishment, but they do not provide information on the population growth and damage potential or temporal population change within a cropping season or year in a given region.

By contrast, the ‘deductive’ approach uses a process-based climatic response model for an species of interest. The benefits of having a process-oriented model based on temperature is that it enables to make predictions for the future taking into account expected climate change as well as variability in cropping seasons or years. Applying this to PYVV transmission by *T. vaporariorum* using the RCP 6.0 climate scenario for 2050, reveals a general reduction of transmission risk in most tropical areas of the world (Fig. 2), whereas the risk increases in temperate regions of the world. The current predictions are made based on annually aggregated monthly average temperatures, this is probably quite accurate for tropical regions of the world, where temperatures do not fluctuate so much throughout the year, but may be less so in more temperate regions of the world. Using predictions based on just the (potato) growing seasons of the year might lead to more exact predictions in those cases. The predictions generated by ILCYM don’t take into account the presence of suitable hosts or extreme continental climates that would influence the ability of the vector to survive and which should be considered when interpreting the maps. Although this can be resolved by masking results to show only risks in areas that are suitable for potato or alternative hosts and/or average monthly temperature are sufficient for vector survival throughout the year, we opted not to apply this as *T.vaporariorum* is polyphagous and suitability maps are not available for all hosts, and insect survival in protected structures in extreme climates is possible. As a result, risk may be shown in areas with extreme climates that need to be interpreted within the local context.

It is likely this same approach can be used to make predictions for other virus vector systems. Experiments on transmission of Banana bunchy top virus (a nanovirus) by its aphid vector *Pentalonia nigronervosa* which is persistently transmitted (in a circulative, non-propagative, non-transovarial manner), also showed a large temperature dependency with inoculation temperature having a larger effect than acquisition temperature (Anhalt and Almeida, 2008). Similarly, clear temperature effects have been shown for transmission of Citrus tristeza virus (a closterovirus) (Bar-Joseph and Loebenstein, 1973), Barley yellow dwarf virus (a luteovirus)(Lowles et al., 1996) and Pea enation mosaic virus (an enamovirus) (Sylvester and Richardson, 1966) by several different aphid species. Our own unpublished results show a temperature dependent transmission of sweet potato leaf curl virus (a begomovirus) by its whitefly vector *Bemisia tabaci,* and Murral et al. (1996) demonstrated that temperature also had a clear effect on Aster yellows phytoplasma transmission by its plant hopper vector. Thus, other pathogen-vector systems are equally sensitive to temperature and similar modeling approach could be used to generate risk maps for them. Options for generating disease transmission indexes for insect vectors have been included in the ILCYM software for this purpose.

In Conclusion, our results show that the combination of insect life parameters and virus transmission functions can be used to effectively identify areas at risk for vector based spread, using *T. vaporariorum* and PYVV as an example. Risk maps generated can be used to guide surveillance, as demonstrated in this study, or pest risk analyses. Bases on the VTI2 maps for PYVV, the virus is predicted to have potential (VTI^S^2 > 2.23) to establish in southern regions of Peru, northern Bolivia, parts of Chile and Argentina, and this potential will increase at larger latitudes in the future, whereas it will decrease in tropical regions. The modeling described in this study was performed using ILCYM v4.0, which has a user-friendly “Shiny” (developed with Shiny software) interface making development of insect phenology models and virus transmission curves extremely easy once the data is entered. Generating maps is equally easy and it should thus provide a versatile tool for researchers to rapidly generate risk maps for insect pest or insect vectored diseases and could be particularly useful to prepare for changes in disease pressure during El Niño years for example. Whereas ILCYM is currently geared at generating risk maps based on monthly weather data, it should relatively easy be modified for local epidemiological simulation and forecasting to provide decision support, even miniaturizing it for smartphone use by farmers and extension workers alike.

## CRediT authorship contribution statement

**H. Gamarra:** Data curation, Investigation, Project administration, Supervision, Writing - original draft, Writing - review & editing. **P. Carhuapoma:** Data curation, Formal analysis, Software, Visualization. **L. Cumapa:** Investigation. **G. González:** Investigation. **J. Muñoz:** Investigation. **M. Sporleder:** Formal analysis, Methodology, Validation, Writing - review & editing. **J. Kreuze:** Conceptualization, Funding acquisition, Methodology, Project administration, Writing - original draft, Writing - review & editing.
