# Supplementary figures and images for: A temperature-driven model for potato yellow vein virus transmission efficacy by Trialeurodes vaporariorum (Hemiptera: Aleyrodidae)
Source: Virus Res. 2020 Nov;289:198109. doi: 10.1016/j.virusres.2020.198109 (PMC7569601; doi:10.1016/j.virusres.2020.198109)

**Supplementary figure 2** Map showing locations of surveyed locations used in this study


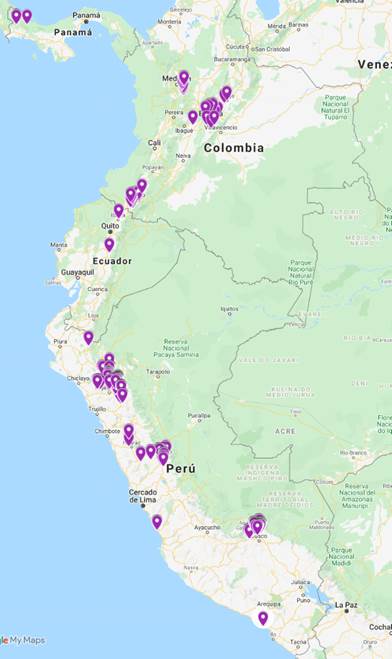

Supplement: Supplementary file 2 [file mmc2.docx]
